# More Biased, Yet More Informed? Documenting Me-Search Stigma Primarily Linked to Researchers’ Own Group Memberships

**DOI:** 10.1177/01461672251339690

**Published:** 2025-06-14

**Authors:** Erdem O. Meral, Corinne A. Moss-Racusin, Jojanneke van der Toorn

**Affiliations:** 1 University of Amsterdam, The Netherlands; 2 Skidmore College, Saratoga Springs, NY, USA; 3 Leiden University, The Netherlands; 4 Utrecht University, The Netherlands

**Keywords:** me-search, queer, stigma, self-relevant research, bias

## Abstract

“Me-searchers” study topics directly or indirectly relevant to themselves—such as social groups they belong to—and are often stigmatized as being more biased (though sometimes more informed) than those researching topics unrelated to their own experience. This study explores how queer and straight researchers are perceived when studying anti-queer bias or other topics through three pre-registered experiments (*N* *=* 823). When both studied anti-queer bias, the queer me-searcher was perceived as more biased, and the straight researcher was perceived as less informed than identical (straight and queer) researchers studying a different topic. Target researchers motivated by social justice (vs. theoretical) implications were also perceived as more biased. Crucially, we identified and tested three conceptual accounts about why and how me-searchers are stigmatized. Supporting the existence of me-search stigma, our results suggest that not only me-searchers but also allied researchers are stereotyped when studying prejudice and discrimination.

Scholars have long debated the potential benefits (e.g., insider perspective, expertise) and drawbacks (e.g., bias and subjectivity, conflicts of interest) of self-relevant research (Brannick & Coghlan, 2007; Chavez, 2015; Hayfield & Huxley, 2015; LaSala, 2003; Merton, 1972; Thorne & Paterson, 2000; Wilkinson & Kitzinger, 2013). However, much of our current understanding of “me-search” comes from the self-reports of me-searchers themselves, with only a few studies offering empirical evidence (Altenmüller et al., 2021; Devendorf et al., 2023; Rios & Roth, 2020; Thai et al., 2021).

Queer researchers studying queer issues often face unwarranted scrutiny and criticism (Eliason, 2016; Harrison & Michelson, 2022; LaSala, 2003; LaSala et al., 2008; Veldhuis, 2022; Yost & Chmielewski, 2013). Not only is their work questioned, but their very interest in the topic is frequently challenged, leading to being labeled—often pejoratively—as “me-searchers.” This term refers to researchers whose work is directly or indirectly relevant to their own identities or experiences, also known as self-relevant (Devendorf et al., 2023) or insider researchers (Hayfield & Huxley, 2015).

In this article, we seek to expand the understanding of me-search stigma, the positive and negative stereotypes associated with conducting self-relevant research. Specifically, we focus on why and how me-searchers, particularly queer me-searchers, are perceived differently from other researchers.

## Understanding (Queer) Me-Search Stigma Is Important

Understanding me-search stigma, particularly as it affects queer researchers, is of practical importance for three key reasons: (a) queer researchers are already subject to prejudice and discrimination, (b) me-search stigma can lead to broader discriminatory practices, and (c) impede free generation of scientific knowledge. Queer individuals in the workforce routinely face discrimination (De Vries et al., 2020) and queer academics are no exception (Bilimoria & Stewart, 2009; J. C. Harris & Nicolazzo, 2020). When queer academics engage in research on queer topics, they encounter additional stressors due to the double marginalization of being queer me-searchers (LaSala et al., 2008; Veldhuis, 2022). Although anecdotal evidence suggests the presence of me-search stigma for queer me-searchers, empirical research on the topic is scarce. Thus, our research specifically focuses on queer researchers to address this gap. The second point concerns the significant backlash me-searchers often face from their peers, which can manifest in various ways, such as encountering unfounded criticism at conferences (such as being accused of having an agenda: Harrison & Michelson, 2022) or during the journal review process (Veldhuis, 2022). Moreover, me-searchers may face challenges at critical junctures in their careers, from graduate school applications (Devendorf, 2020) to securing funding (Carnethon et al., 2020; Hoppe et al., 2019; Prock et al., 2019), and even during promotion and tenure decisions (Gardner et al., 2017; J. L. Harris, 2020). Finally, me-search stigma can impede scientific progress in several ways. For one, me-search stigma can discourage scholars investigating topics such as discrimination and marginalization, topics that are more frequently studied by scholars from marginalized communities (Hoppe et al., 2019). Consequently, discouraging diverse voices may create a more homogeneous group of researchers (Biernat et al., 2020), which is detrimental to scientific thought and progress (Intemann, 2009; Page, 2007). Taken together, we argue that understanding the root causes of me-search stigma is crucial for eliminating these discriminatory barriers for all me-searchers and ensuring healthy scientific progress.

## Stereotypes Associated With Me-Search Stigma

### Me-Search and Bias

One way in which me-search stigma manifests itself is through perceptions of bias. Personal accounts from me-searchers reveal that they are often seen by others as more partial and less credible than researchers who do not engage in self-relevant research (Devendorf, 2020; Eliason, 2016; Gardner et al., 2017; Harrison & Michelson, 2022; Hendrix, 2002; LaSala et al., 2008; Oh, 2019; Veldhuis, 2022; Yost & Chmielewski, 2013). These anecdotal experiences are corroborated by a few empirical studies, showing that me-searchers are indeed perceived as more biased compared to researchers who are not personally connected to their topics both by other researchers and the public (Altenmüller et al., 2021; Devendorf et al., 2023; Rios & Roth, 2020; Thai et al., 2021). Taken together, these insights suggest that me-search stigma often involves stereotyping me-searchers as more biased than other researchers.

### Me-Search and Informedness

Despite the bias stereotype, me-searchers, particularly those from marginalized groups, often challenge the notion that me-search is primarily associated with bias and less rigorous science. They argue that self-relevant research can provide unique, valuable insights, due to the researcher’s first-hand experience with the topic (Amabile & Hall, 2021; Collins & McNulty, 2020; LaSala, 2003; Moss-Racusin, 2021; Oh, 2019; Thorne & Paterson, 2000). Furthermore, experimental research suggests that self-relevance can actually enhance public perceptions of a researcher’s expertise (Thai et al., 2021), as has also been shown for journalists (Torrez et al., 2024) and activists (Wallace et al., 2024). Thus, while me-search stigma may involve negative stereotypes of bias, it can also lead to a more positive stereotype: the perception that me-searchers are more informed than other researchers.

## Why Is Me-Search Stigmatized?

From our review of past work on the topic, we identified three conceptual accounts, for current purposes labeled the (a) shared group membership account, (b) social justice account, and (c) prejudice account. These accounts guided our predictions about how me-searchers would be perceived relative to other researchers. In grouping past work under these conceptual accounts and drawing predictions accordingly, we aimed to build on past work and contribute to research on me-search by theorizing and systematically testing why and how me-search is stigmatized.

### The Shared Group Membership Account

The *shared group membership account* posits that queer me-search is stigmatized because me-searchers are researching their ingroup. This aligns with positivist (or naïve empiricist) critiques of me-search, which argue that insider status compromises objectivity (Breen & Darlaston-Jones, 2010; Gardner et al., 2017; Teo, 2018) by making the subject matter personal. Me-searchers themselves acknowledge these risks, noting that shared group membership might lead to assumptions of common knowledge with participants, potentially reducing the rigor of inquiry (Hayfield & Huxley, 2015; Kanuha, 2000). However, shared group membership can also provide advantages, such as easier access to participants (LaSala, 2003; Maykovich, 1977; Platzer & James, 1997) and an increased understanding of the group’s issues and perspectives (Dwyer & Buckle, 2009; LaSala, 2003). Taken together, the shared group membership account would suggest that me-search stigma is driven by the insider status of researchers within the communities they study.

### The Social Justice Account

Beyond mere group membership, the *social justice account* suggests that me-searchers are stigmatized because their research often has social justice implications. In this view, me-search is not just about knowledge production but also about advocacy or activism. Critics argue that me-searchers have a personal agenda (Thai et al., 2021), viewing them as activists rather than “true” researchers; for instance queer me-searchers might be dismissed as “some kind of gay activist” rather than serious scholars (Harrison & Michelson, 2022, p. 759). Some me-searchers embrace this, acknowledging that their work is intertwined with their commitment to justice (Eliason, 2016). This stigma may be explained by system-justification theory, which suggests that efforts to challenge and dismantle marginalization can threaten the status quo (Jost & Van der Toorn, 2012). When researchers from marginalized groups—whether focusing on Black individuals (J. L. Harris, 2020), women (Moss-Racusin, 2021), or queer people (Veldhuis, 2022)—seek to understand and address inequality, their efforts may be met with resistance because they challenge the status quo. Stigmatizing me-search as biased can thus serve to discredit these efforts and diminish their potential impact, thereby maintaining the status quo.

### The Prejudice Account

Lastly, the *prejudice account* explores whether me-search stigma against queer researchers is rooted primarily in broader biases against queer individuals, regardless of their research topic or methodology. Not all groups are equally valued in the scientific community, and queer professionals, particularly in STEM fields, often face significant barriers to career advancement. Compared to their straight counterparts, queer professionals in science are more likely to be professionally devalued—for example, being perceived as less skilled—by colleagues (Cech & Waidzunas, 2021, 2022). At the same time, some occupations are seen as more fitting for queer people than they are for straight people (Palmer et al., 2022; Stokes, 2015). Combined, the prejudice account would suggest that a queer me-searcher faces stigma not primarily because of their research focus but because of their identity as a queer researcher.

### Past Experimental Evidence

Several experimental studies have explored the perceptions of me-search, though these investigations often touched on the underlying accounts only partially and not always explicitly. For instance, surveying clinical, counseling, and school psychology researchers in the United States, Devendorf et al. (2023) observed that disclosure of me-search on suicide, schizophrenia, depression, and cancer were associated with both positive (e.g., motivated and admirable) and negative stereotypes of the researcher (e.g., biased and selfish) compared to disclosure of non-self-relevant research.

In another vignette experiment, Altenmüller et al. (2021) explored public perceptions of a queer researcher investigating workplace experiences of queer employees. Participants rated either a queer researcher studying anti-queer bias (a me-searcher) or a straight researcher examining the same topic (not a me-searcher). Their findings showed that people with positive attitudes toward queer individuals rated the queer me-searcher as more credible than the straight researcher. However, because sexual orientation and me-searcher status were conflated—queer researchers were always me-searchers and straight researchers never were—the design leaves open the question of whether the observed biases exhibited were mainly associated with the researcher’s me-searcher status (supporting the shared group membership account) or due to general prejudice against queer individuals (supporting the prejudice account).

In a study, Thai et al. (2021) examined public perceptions of me-searchers studying various prejudices, including weight stigma, racism, and ageism using bogus researcher profiles with photos. The authors compared the perceptions of me-searchers (e.g., a Black researcher studying racism) with those of researchers studying prejudice from other perspectives (e.g., a Black researcher studying ageism). The results indicated that me-searchers were perceived as more trustworthy due to their first-hand experience but also less trustworthy because they were seen as motivated by vested interest. This comparison helps distinguish between shared group membership and social justice accounts, as effects in control conditions were smaller or absent, supporting the shared group membership account. However, target researchers in both experimental and control conditions in Thai et al. (2021) conducted research with clear social justice implications (e.g., racism vs. ageism), and no comparison was made between researchers from marginalized communities when they conduct prejudice research and non-prejudice research. We argue that to effectively disentangle these three accounts, research should compare me-searchers to researchers whose work has less apparent social justice implications while also manipulating their group identity.

## Current Research

Overall, past evidence suggest that me-search stigma may arise from a researcher’s focus on their ingroup (i.e., the shared group membership account), the potential social justice implications of their work (e.g., the social justice account), or simply the researcher’s identity (i.e., the prejudice account). Building on these insights, our current research aimed to investigate public perceptions of queer me-searchers and asked whether they would be perceived as more biased yet more informed than other researchers, whether straight or queer. For brevity, in the remainder of the article, we will refer to queer researchers studying anti-queer bias as “queer me-searchers.”^1^ We did not have specific a priori predictions about which theoretical account would most strongly explain me-search stigma. In addition to examining perceived bias and informedness, we also explored other outcomes, including perceived quality of the researchers’ work, perceived appropriateness of their research, perceived personal benefit of conducting such research, overall impressions of the researcher, and the moderating role of pre-existing attitudes toward queer individuals.

We conducted three experiments where we systematically varied the researcher’s sexual orientation (queer vs. straight in Studies 1–3), gender (male vs. female in Study 1), research topic (attentional bias vs. anti-queer bias in Study 2), and research approach (advocacy vs. neutrality in Study 3). In all three studies, we measured public perceptions of researchers as understanding the public’s perceptions of and attitudes toward science is important for making effective policy recommendations and science communication in general (Bromme & Goldman, 2014; Field & Powell, 2001; Sinatra & Hofer, 2016).

The design, materials, sample sizes, confirmatory and exploratory questions, and analysis plans were pre-registered prior to data collection for both studies (to access pre-registration documents for Study 1, see: https://aspredicted.org/X82_DCG, for Study 2, see: https://aspredicted.org/ZBC_XST, for Study 3, see: https://aspredicted.org/Y3D_S9G), with further documentation in our OSF page (https://osf.io/8h47r/). We report all manipulations, measures, and exclusions in these studies (with some exploratory tests reported as Supplemental Information at https://osf.io/8h47r/).

## Pre-Testing Names

We manipulated the researcher’s sexual orientation and gender by changing their and their partner’s name. We first pretested 10 names on perceived gender, competence, warmth, and recognizability, selecting those distinctly viewed as male or female but similar on other dimensions (see https://osf.io/jtmbz).

## Study 1

In Study 1, we compared how queer me-searchers and straight researchers are perceived when both study anti-queer bias, serving as a conceptual replication of Altenmüller et al. (2021). Because perceptions of sexual orientation are gendered (Freeman et al., 2010), and gay men and lesbian women may face distinct consequences for being queer (Ahmed et al., 2013; Aksoy et al., 2019), we extended Altenmüller et al. (2021) by exploring how researcher gender moderates these perceptions.

*Confirmatory Hypotheses*: Queer researchers are perceived as more biased (H1) and more informed (H2) than their straight counterparts who do the same research.
*Research Question*: Does researcher gender moderate the effect of target researcher sexual orientation on perceived bias and informedness?

### Methods

#### Participants and Design

This was a 2 (Researcher Gender: Male vs. Female) × 2 (Researcher Sexual Orientation: Queer vs. Straight) between-subjects design, with all target researchers studying anti-queer bias. Based on our a priori determined sample size, we recruited 358 UK participants (≥18 years) via Prolific (Peer et al., 2017) and paid them €2.00. We excluded participants who failed our pre-registered inclusion criteria (*n* *=* 19; for more information see the Supplemental Information at https://osf.io/bdkre) and—by deviating from our pre-registration—those who failed manipulation checks^2^ (*n* = 104), leaving the final sample shown in Table 1. All participants gave informed consent.

**Table 1.** Sample Characteristics for Studies 1 to 3.

Variables	Study 1	Study 2	Study 3
Age *M* (*SD*)	40.62 (14.47)	39.30 (13.05)	42.90 (13.40)
Women	104	129	152
Men	106	139	164
Self-described	4	2	2
Straight	198	233	281
Bisexual	27	17	19
Gay	9	8	9
Lesbian	3	6	3
Self-described	4	5	5
Sample size	235	270	318

*Note.* Further details about participants who self-described for gender and sexual orientation can be found in Supplemental Information at https://osf.io/bdkre.

#### Procedure and Measures

Participants read a forum post—ostensibly selected from a larger pool of posts—and evaluated the researcher on the forum post and their work on various dimensions. In the post adapted from prior work (Correll et al., 2021), a graduate student (either Daniel or Jennifer) talks about how they are studying anti-queer bias and that they are looking for an apartment with their partner (either Noah or Sophia), see Appendix A.1. After reading the forum post, participants answered comprehension checks, completed the six outcome measures in a random order (with items randomized as well), and indicated their attitudes. Finally, participants answered several manipulation checks, completed demographics questions, and were debriefed and compensated.

Information about measures (e.g., example items, reliability scores, and sources) can be found in Table 2 with additional information (also about demographics, attention, and comprehension checks) as Supplemental Information (https://osf.io/bdkre).

**Table 2.** Measures Used Across All Three Studies With Example Items and Reliability Scores.

Dependent variables			
Construct (number of items)	Example items	Reliability	Study
Perceived bias (6)	To what extent do you think Daniel* is an objective researcher?	$\alpha_{1}$ = .79, $\alpha_{2}$ = .83, * $\alpha_{3}$ * = .81	1, 2, 3
Perceived informedness (4)	*Daniel is very well-informed with regard to his research topic*	$\alpha_{1}$ = .73, $\alpha_{2}$ = .71, * $\alpha_{3}$ * = .79	1, 2, 3
Perceived personal benefit (5)	*To what extent do you think that Daniel stands to personally benefit from his research?*	$\alpha_{1}$ = .82, $\alpha_{2}$ = .84, $\alpha_{3}$ = .86	1, 2, 3
Perceived appropriateness (5)	*I think that Daniel’s research topic is a good fit for him*	$\alpha_{1}$ = .72, $\alpha_{2}$ = .76, $\alpha_{3}$ = .75	1, 2, 3
Perceived quality of work (9)	*Daniel likely conducts high-quality research*	$\alpha_{1}$ = .95, $\alpha_{2}$ = .94	1, 2
Anticipated questionable research practices (4)	*To what extent do you think Daniel would fail to disclose all potentially relevant conflicts of interest?*	*α* = .93	3
Manipulation check questions			
Construct	Questions and answer options		Study
Target researcher’s gender	*Q: What was the graduate student’s gender?* *A: “Male,” Female,” “I don’t remember”*		1, 2, 3
Partner’s gender	*Q: What was the gender of the graduate student’s partner?* *A: “Male,” Female,” “I don’t remember”*		1, 2, 3
Research topic	*Q: What was the graduate student’s area of research?* A: *“Attentional bias and social perception.” “Bias against LGBTQ+ individuals.” “Animal welfare.” “Social exclusion.” “I don’t remember.”*		1, 2, 3
Research approach	*Q: According to the graduate student, what was the main driver of their research?* *A: “Social justice implications of research and advocacy.” “Theoretical implications of research and neutrality.” “I don’t remember.”*		3

*Note.* Items measuring perceived bias, informedness, personal benefit, appropriateness, and quality of work were adapted from past work on the topic (Correll et al., 2021). Items pertaining to questionable research practices were adapted from work on questionable research practices (Fiedler & Schwarz, 2016; Fiedler et al., 2018). All dependent variables were measured on a 7-point Likert-type scale ranging from 1 (*not at all*) to 7 (*very much*).

* For ease of reading, here we refer to target researcher as Daniel. Yet, in Study 1, the correct name and accompanying pronouns were piped into each question depending on the experimental condition.

### Results and Discussion

For all analyses, we conducted separate 2 (Researcher Sexual Orientation: Queer vs. Straight) by 2 (Researcher Gender: Male vs. Female) between-subjects ANOVAs where we effect coded both the sexual orientation (queer = −0.5, straight = 0.5) and the gender factor (female = −0.5, male = 0.5). The descriptive statistics for all outcome measures can be found in Table 3.

**Table 3.** Descriptive Statistics and Analysis Results for Study 1.

Descriptive statistics and post hoc comparisons				
Dependent variable	Queer		Straight	
	Male, *M* (*SD*)	Female, *M* (*SD*)	Male, *M* (*SD*)	Female, *M* (*SD*)
Bias	4.43 (1.04)_a_	4.26 (0.86)_a_	3.30 (0.87)_b_	3.53 (0.92)_b_
Informedness	4.95 (1.10)_a,c_	5.01 (0.84)_a_	4.45 (1.03)_b_	4.50 (0.84)_b,c_
Personal benefit	5.15 (1.02)_a_	5.28 (0.89)_a_	4.04 (1.09)_b_	4.07 (1.20)_b_
Appropriateness	5.00 (1.05)_a,c_	5.16 (0.81)_a_	4.71 (0.90)_a_	4.69 (1.09)_b,c_
Quality of work	4.50 (1.44)_a_	4.86 (1.10)_a_	4.91 (1.20)_a_	4.70 (1.37)_a_
Results of the planned ANOVAs				
Dependent variable	Independent variable	*F*(1,231)	*p*	$\eta_{p}^{2}$
Bias	S.O.	59.63	<.001	.205
	Gender	0.04	.83	<.001
	S.O. × Gender	2.80	10	.012
Informedness	S.O.	18.92	<.001	.076
	Gender	0.07	.42	.003
	S.O. × Gender	0.17	.68	.001
Personal benefit	S.O.	70.36	<.001	.233
	Gender	0.29	.59	.001
	S.O. × Gender	0.13	.73	.001
Appropriateness	S.O.	8.94	.003	.037
	Gender	0.32	.57	.002
	S.O. × Gender	0.52	.47	.001
Quality of work	S.O.	0.56	.46	.002
	Gender	0.21	.65	.001
	S.O. × Gender	2.93	.09	.013

*Note.* At the top of the table, the values that do not share subscript within a row are statistically different from each other (Tukey adjusted *p* values, <.05). Only the results pertaining to perceived bias and informedness are confirmatory. S.O. = sexual orientation of the target researcher; Gender = gender of the target researcher.

**Figure 1.**
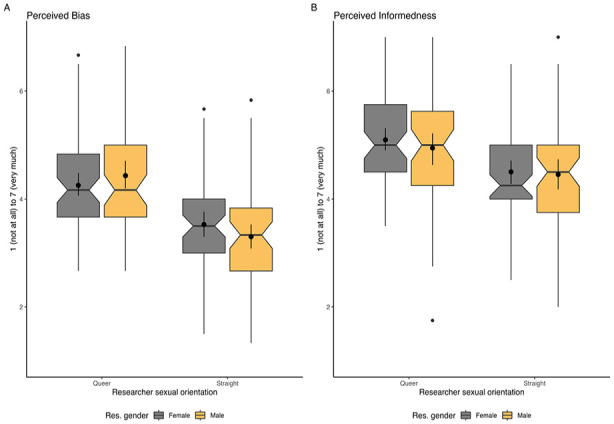
This is a notched box plot of the confirmatory results in Study 1. The box goes from the first quartile (25th percentile at the lower end) to the third quartile (75th percentile at the top), namely the interquartile range (IQR). The whiskers extend to 1.5 × IQR on both ends. The line at the center of the box plot represents the median with the notches representing the 95% CI of the median. The black dot represents the mean value with the lines coming out representing the 95% CI of the mean. (A) Perceived bias and (B) perceived informedness.

In line with H1, there was a significant main effect of researcher sexual orientation, such that participants perceived queer researchers as more biased than straight researchers, see Figure 1A and Table 3. Neither gender nor the interaction of gender and sexual orientation had a significant effect on perceived bias. Consistent with H2, we observed a significant main effect of researcher sexual orientation on perceived informedness, see Figure 1B and Table 3. Specifically, and in line with H2, participants perceived queer researchers as more informed than straight researchers. Like perceived bias, neither gender, nor the interaction of gender and sexual orientation, had a statistically significant effect on perceived informedness.

Furthermore, participants thought that it was more appropriate and personally beneficial for queer me-searchers (vs. straight researchers) to investigate anti-queer bias. We observed no significant differences in terms of perceived quality of work across conditions. Overall, although offering initial support for me-search stigma and replicating previous work (Altenmüller et al., 2021), the design of Study 1 did not allow us to tease out if this stigma largely stemmed from doing research into one’s ingroup (i.e., the shared group membership account) or a general negativity toward queer researchers (i.e., the prejudice account). This is because in Study 1 (similar to Altenmüller et al., 2021), the me-searcher status is confounded with sexual orientation such that queer researchers were always me-searchers and straight researchers were not.

## Study 2

To overcome this major shortcoming, we designed Study 2 to rule out the alternative explanation that me-search stigma is stigma against queer individuals in disguise. To do so, we separately manipulated the sexual orientation of the researchers (queer vs. straight) and their research topic (anti-queer bias vs. attentional bias). This allowed us to tease out if me-search stigma is largely associated with a prejudice account (i.e., queer researchers face biased perceptions) or a shared group membership account (i.e., queer researchers face biased perceptions only when they do me-search).

*Confirmatory hypothesis 1*: Queer me-searchers will be perceived as more biased (H1a) and more informed (H1b) than straight researchers who study anti-queer bias—*replication of Study 1.*
*Confirmatory hypothesis 2*: Queer me-searchers will be perceived as more biased (H2a) and more informed (H2b) than queer researchers who study attentional bias. (Alternatively, we may observe no difference between the two if findings of Study 1 were mostly about a negative evaluation of queer researchers—*the prejudice account*.)

In Study 1, we did not observe a significant moderating effect of researcher gender.

### Methods

This was a two-way study with two between-subject factors (2 [research topic: anti-queer bias vs. attentional bias] by 2 [researcher sexual orientation: queer vs. straight]) with random assignment to conditions. In this study, the target researcher was always a man—this decision was motivated by the effects being slightly larger for the male (vs. female) researcher. Based on our a priori determined sample size (for the power simulation, see https://osf.io/8h47r/), we recruited 403 participants online (screening criteria: current U.K. residents, over the age of 18) via Prolific UK (Peer et al., 2017)—payment: €2.00. Like Study 1, we first excluded participants based on our pre-registered exclusion criteria (*n* *=* 21), then—deviating from our pre-registration—we excluded participants who failed the manipulation checks (*n* = 110).^3^ The sample characteristics of the final sample are in Table 1. All participants provided informed consent before starting the study.

The procedure was identical to Study 1 with one major exception: the manipulation of the research topic in the forum posts (research into anti-queer bias or attentional bias; for the full post, see Appendix A.2). We used the same items as in Study 1 for dependent variables, comprehension, manipulation, and attention checks, and demographics (see Table 2 and Supplemental Information at https://osf.io/bdkre).

### Results and Discussion

As in Study 1, we ran ANOVAs (categorical factors were effect coded: sexual orientation [queer = −0.5, straight = 0.5] and research topics [anti-queer bias = −0.5, attentional bias = 0.5]). Descriptive statistics for all the outcome variables and the results of these analyses can be found in Table 4.

**Table 4.** Descriptive Statistics and Analysis Results for Study 2.

Dependent variable	Queer researcher *M* (*SD*)		Straight researcher *M* (*SD*)	
	Anti-queer bias	Attentional bias	Anti-queer bias	Attentional bias
Bias	4.12 (0.84)_a_	3.50 (0.57)_b_	3.36 (0.77)_b_	3.33 (0.75)_b_
Informedness	5.11 (0.98)_a_	5.01 (0.79)_a_	4.27 (0.89)_b_	4.97 (0.83)_a_
Personal benefit	5.09 (0.96)_a_	4.73 (0.99)_a_	3.89 (1.11)_b_	4.54 (0.91)_c_
Appropriateness	5.20 (1.03)_a_	5.51 (0.81)_a_	4.41 (1.21)_b_	5.50 (0.84)_a_
Quality of work	4.84 (1.16)_a_	5.16 (0.83)_a_	4.77 (1.16)_a_	4.93 (0.83)_a_
Results of the planned analyses				
Dependent variable	Independent variable	*F*(1,266)	*p*	$\eta_{p}^{2}$
Bias	S.O.	27.05	<.001	.092
	Topic	12.98	<.001	.047
	S.O. × Topic	10.97	.001	.040
Informedness	S.O.	16.83	<.001	.060
	Topic	7.72	.006	.028
	S.O. × Topic	14.36	<.001	.051
Personal Benefit	S.O.	32.82	<.001	.110
	Topic	1.42	.24	.005
	S.O. × Topic	17.18	<.001	.061
Appropriateness	S.O.	11.12	<.001	.041
	Topic	34.68	<.001	.116
	S.O. × Topic	10.78	<.001	.039
Quality of work	S.O.	1.58	.21	.006
	Topic	3.81	.05	.010
	S.O. × Topic	.40	.53	.002

*Note.* Within a row, the values that do not share a subscript are significantly different from each other (<.05, Tukey adjusted *p* values). Only the results pertaining to perceived bias and informedness are confirmatory. S.O. = sexual orientation of the target researcher (queer vs. straight); Topic = The research topic of the target researcher (anti-queer bias vs. attentional bias).

**Figure 2.**
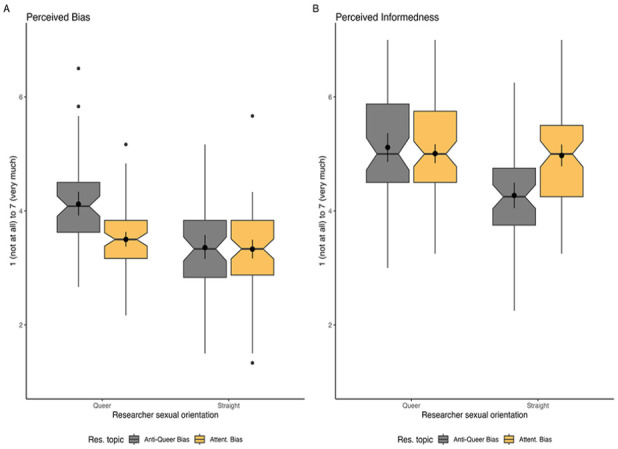
This is a notched box plot of the confirmatory results in Study 2. See Figure 1 for the description of the notched box plot graph. (A) Perceived bias and (B) perceived informedness.

There was a significant main effect of researcher sexual orientation on perceived bias such that queer researchers were perceived to be more biased than straight researchers. There was also a significant main effect of researcher topic where researchers studying anti-queer bias were perceived as more biased than researchers who studied attentional bias. More importantly, we also observed a significant interaction effect. Pairwise comparisons (Tukey) showed that participants rated the queer researcher who studied anti-queer bias as more biased than the queer researcher who studied attentional bias (*t*(266) = 4.96, *p* < .001, *d* = 0.85), the straight researcher who studied anti-queer bias (*t*(266) = 5.66, *p* < .001, *d* = 1.05), and the straight researcher who studied attentional bias (*t*(266) = 6.23, *p* < .001, *d* = 1.08). The perceived bias ratings of the other three researchers were not significantly different from each other (*p*s > .47). Thus, participants perceived the queer me-searcher to be more biased than any other target researcher in the study. This pattern of results replicates and extends Study 1’s findings and offers support to Hypotheses 1a and 2a. The visualization can be seen in Figure 2A. These results offer more support for the shared group membership account compared to the prejudice account because the queer researchers were only stigmatized when they conducted me-search.

We observed a statistically significant main effect of researcher sexual orientation and research topic on perceived informedness. Participants rated the queer researchers as more informed than straight researchers and perceived the researchers who studied anti-queer bias (vs. attentional bias) as more informed. We followed up the statistically significant interaction effect with post hoc tests (Tukey). Supporting H1b, the queer me-searcher was perceived as more informed than the straight researcher when they both studied anti-queer bias, *t*(265) = 5.23, *p* < .001, *d* = .97. However, the queer me-searcher was not perceived as more informed than the queer researcher who studies attentional bias, *t*(265) = .73, *p* = .89, *d* = 0.12, failing to support H2b. Instead, as can be seen in Figure 2B, the informedness difference seems to be driven by the lower informedness ratings of the straight researcher who studied anti-queer bias. This researcher was perceived as less informed than the queer and straight researchers who studied attentional bias (queer researcher: *t*(265) = −4.88, *p* < .001, *d* = 0.85; straight researcher: *t*(265) = −4.59, *p* < .001, *d* = 0.82). The other comparisons were not statistically significant (*p* > .78). These results suggest that me-searchers are not the only ones who are penalized for doing research into marginalized communities, allied researchers—straight researchers studying anti-queer bias—are also penalized.

In our exploratory analyses, the queer me-searcher was perceived to benefit more from their work on anti-queer bias than the straight researcher. It was also seen as more appropriate for the queer me-searcher to conduct such research. Importantly, the straight researcher was viewed as benefiting the least and being the least appropriate to study anti-queer bias. Replicating Study 1, there were no significant effects on perceived quality of work. These results, like our confirmatory findings, show that both queer me-searchers and allied straight researchers are penalized for studying in anti-queer bias—but in different ways.

## Study 3

The fact that both queer me-searchers and allied straight researchers are penalized for studying anti-queer bias could ultimately help to maintain and reinforce the status quo (Jost & Van der Toorn, 2012). Although offering a strong starting point, results of Studies 1 and 2 cannot yet adequately address if queer me-searchers are perceived negatively due to conducting queer research while being queer (*the shared group membership account*) or conducting research with a more explicit goal to mitigate discrimination and prejudice faced by queer individuals (*social justice account*). To examine these two accounts, in Study 3 we ask: Does a specific focus on social justice implications of one’s work influence bias and informedness perceptions of queer me-searchers?

Based on our previous results, we tested two theoretical accounts with respect to perceived bias. We argue that the shared group membership account would receive more support if queer (vs. straight) researchers are perceived as biased regardless of their emphasis on neutrality (vs. advocacy). On the other hand, we argue that the social justice account would receive more support if researchers who emphasize the social justice implications of their work (vs. neutrality) are perceived as biased regardless of their sexual orientation. Based on our focal test, we expected an interaction between these accounts such that both queer and straight researchers would be perceived as less biased when explicitly expressing neutrality (vs. advocacy). However, this effect would be weaker for queer researchers, as they may be perceived as advocates by default.

### Method

#### Participants and Design

This is a 2 (Researcher Sexual Orientation: Queer vs. Straight) by 2 (Researcher Approach: Neutrality vs. Advocacy) between-subjects design. There were 502 completed surveys^4^ out of which we excluded participants based on our pre-registered exclusion criteria of failing at least two attention checks (*n* = 0)^5^ or manipulation checks about target gender (*n* = 18), or the target sexual orientation (*n* = 40), or their research approach (*n* = 126). Characteristics of the final sample are in Table 1. All participants provided informed consent before starting the study.

#### Procedure and Materials

The procedure of the study was near identical to previous studies. Participants read forum posts and evaluated the target researcher where all targets (Daniel) studied anti-queer bias. We manipulated research approach, which either focused on neutrality or advocacy (see Appendix A.3 for the full forum post).

After reading the forum post, participants answered questions about the researcher and their work. Instead of asking the researcher’s quality of work as in Study 2, here we asked about whether participants anticipated the researchers to engage in questionable research practices (QRPs; see Table 2 and Supplemental Information). For the rest, we used the same items.

### Results and Discussion

Using the same strategy as in Study 1, we conducted ANOVAs and effect-coded the sexual orientation (queer = −0.5, straight = 0.5) and research approach (advocacy = −0.5, neutrality = 0.5) factors. Descriptive statistics for all the dependent variables and the statistical results of these analyses can be found in Table 5.

**Table 5.** Descriptive Statistics and Analysis Results for Study 3 for All Dependent Variables.

Descriptive statistics and post hoc comparisons				
Dependent variables	Queer researcher *M* (*SD*)		Straight researcher *M* (*SD*)	
	Advocacy	Neutrality	Advocacy	Neutrality
Bias	4.17 (0.95)_a_	3.94 (0.99)_a,b_	3.65 (1.12)_b_	3.03 (0.95)_c_
Informedness	5.18 (1.04)_a_	5.04 (0.94)_a_	4.31 (1.13)_b_	4.50 (0.93)_b_
Personal benefit	5.29 (1.02)_a_	4.89 (0.89)_a_	4.17 (1.16)_b_	3.73 (1.28)_b_
Appropriateness	5.34 (0.92)_a_	5.07 (1.02)_a,b_	4.71 (1.02)_b_	4.96 (0.98)_a_
QRPs	2.54 (1.33)a	2.38 (1.24)_a_	2.49 (1.52)_a_	2.29 (1.15)_a_
Results of the planned analyses				
Dependent variables	Independent variables	*F*(1,314)	*p*	$\eta_{p}^{2}$
Bias	S.O.	40.37	<.001	.114
	Approach	13.95	<.001	.043
	S.O. × Approach	2.97	.09	.009
Informedness	S.O.	38.66	<.001	.110
	Approach	.06	.81	<.001
	S.O. × Approach	2.15	.14	.007
Personal benefit	S.O.	87.15	<.001	.217
	Approach	11.62	<.001	.036
	S.O. × Approach	.03	.86	<.001
Appropriateness	S.O.	11.05	<.001	.034
	Approach	.01	.91	< .001
	S.O. × Approach	5.45	.02	.017
QRPs	S.O.	.17	.68	.001
	Approach	1.50	.22	.005
	S.O. × Approach	.03	.86	<.001

*Note.* In the top part of the table, values that do not share a subscript within each row are significantly different from each other (<.05, Tukey adjusted post hoc tests). We only had confirmatory hypotheses about perceived bias. S.O. = sexual orientation of the target researcher (queer vs. straight); Approach = research approach of the target researcher (advocacy vs. neutrality); QRPs = Questionable research practices.

**Figure 3.**
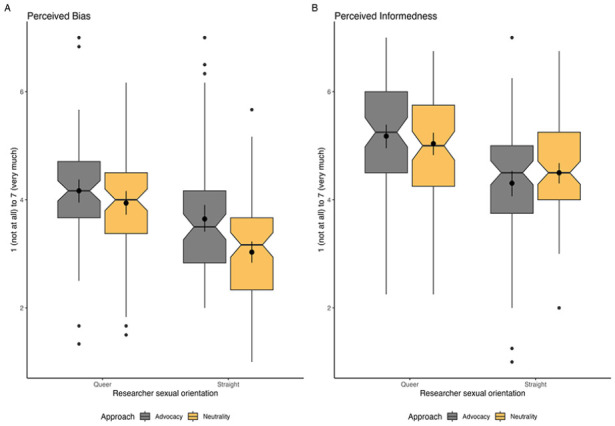
This is a notched box plot of the confirmatory and exploratory results in Study 3. See Figure 1 for the description of the notched box plot graph. (A) Perceived bias and (B) perceived informedness.

Queer (vs. straight) and advocate (vs. neutral) target researchers were perceived as more biased (see Figure 3A). Even though the predicted interaction effect was not statistically significant, a series of post hoc tests (Tukey) revealed that while queer me-searchers were not perceived more or less biased depending on their research approach, *t*(314) = 1.40, *p* = .49, *d* = 0.23, the straight researcher with a neutrality approach was perceived as less biased than the straight researcher with an advocacy approach, *t*(314) = 3.92, *p* < .001, *d* = 0.61. The straight researcher with a neutrality approach was also perceived as less biased than queer researchers with an advocacy, *t*(314) = 5.23, *p* < .001, *d* = 0.97, and a neutrality approach, *t*(314) = 5.23, *p* < .001, *d* = 0.97—effectively making them the target researcher perceived to be least biased of the four possible target researchers.

Exploratory analyses replicated Studies 1 to 2 and suggested that queer researchers were perceived as more informed than straight researchers (see Figure 3B and Table 5), with no other significant effects. This suggests that having a justice- or theory-oriented approach is mainly related to bias but not informedness perceptions. Other exploratory variables showed a similar pattern such that the main driver of the observed differences was the sexual orientation of the target researcher. We did not observe any statistically significant effects on anticipated QRPs, resembling the findings we observed in previous studies with regard to perceived quality of work ratings. Taken together, these results suggest a nuanced picture where shared group membership and social justice implications both differentially influence perceptions of me-search and research into anti-queer bias by straight researchers.

## General Discussion

Across three studies, we investigated how and why me-search stigma influences public perceptions of queer and straight researchers, testing three theoretical accounts: the shared group membership account (i.e., me-search stigma stems from the researcher studying their own group), the social justice account (i.e., me-search stigma stems from perceptions that researchers are motivated by advocacy rather than science), and the prejudice account (i.e., me-search stigma stems from broader negative bias against queer people, regardless of their research topic).

Consistent with previous research (Altenmüller et al., 2021; Thai et al., 2021), we found that me-search stigma was associated with stereotypes, particularly perceptions of increased bias. Further, we found support for the shared group membership account (Studies 2 and 3) and the social justice account (Study 3), while the prejudice account received relatively less empirical support (Study 2). Specifically, queer me-searchers were perceived as both more biased and more informed than straight researchers when both studied anti-queer bias (Studies 1–3). This pattern aligns with previous empirical (Devendorf et al., 2023; Thai et al., 2021) and anecdotal evidence (Hayfield & Huxley, 2015; LaSala et al., 2008) suggesting that me-search stigma is associated with both negative and positive stereotypes. Furthermore, our findings lend stronger support to the shared group membership account over the prejudice account, as the queer researchers studying attentional bias did not experience these same stereotypes (Study 2). However, the picture is more nuanced. Contrary to our expectation that queer me-searchers are perceived as more informed, the results indicate that it is the straight researcher studying anti-queer bias who is perceived as the least informed (see Figure 2). Thus, while shared group membership does seem to drive certain stereotypes associated with me-search stigma, the overall narrative is more complex than a straightforward “positive bias.”

We argue that the results observed in Study 2 suggest that me-search stigma may serve a system-justifying function (Jost & Van der Toorn, 2012). Both the queer me-searcher and the allied (straight) researcher encountered negative stereotypes: the queer me-searcher was perceived as biased, while the straight researcher was seen as less informed. This dual stigmatization implies that if both groups are penalized for studying anti-queer bias, the societal impact of such research may never materialize—maintaining the status quo. We explored this possibility in Study 3, where queer me-searchers were perceived as more biased than straight researchers, supporting the shared group membership account and echoing self-reports of me-searchers (Gardner et al., 2017; LaSala et al., 2008). The social justice account only received partial support because while research with social justice (vs. theoretical) implications was perceived as more biased (in line with anecdotal evidence, Eliason, 2016; Harrison & Michelson, 2022), me-searcher status did not influence this perception. This pattern of results extends the ideas around vested interest proposed by Thai et al. (2021) such that straight researchers who frame their motivation as advocacy are also perceived as more biased. In this case, their vested interest stems from their concern for the social justice implications of their work.

Importantly, the pattern of means and post hoc tests aligned with the predictions of the social justice account, suggesting that these two accounts might be more intertwined than we initially anticipated. Being part of a marginalized group may naturally align a researcher’s interests with the group’s broader social justice goals (also see Saguy et al., 2020). To further disentangle these accounts, future research could focus on topics related to queer experiences that have fewer overt social justice implications, such as queer romantic relationships. This approach could help isolate the effect of social justice implications from the impact of group membership on perceptions of bias.

In comparing me-searchers to allied straight researchers who explicitly indicated social justice motivations, we bridge two previously distinct literatures: research on science perception and on ally perception. Reflecting prior findings regarding straight allies to queer individuals (Goldstein, 2017; Rostosky et al., 2015), our results show that allied researchers also face both advantages (e.g., not perceived as biased) and disadvantages (e.g., perceived as less informed) when examining anti-queer bias. Interestingly, straight allies themselves emphasize increased knowledge as a benefit of allyship (Rostosky et al., 2015), contrasting public perceptions observed in our study. Future research should further explore perceptions of me-searchers and allied researchers to deepen our understanding of how group membership and allyship relate to the perception of science and scientists more broadly.

We observed less support for the prejudice account—the idea that queer researchers may face negative stereotypes regardless of their topic or approach, similar to the challenges queer researchers in STEM fields experience (Cech & Waidzunas, 2021, 2022). In Study 2, only the queer me-searcher was perceived as more biased and not the queer researcher studying attentional bias. This suggests that the stereotype is mainly associated with me-search but not being a queer scientist in general. However, we do not believe that me-search stigma is independent from prejudices, particularly when considering the role of dispositional prejudices individuals may hold. Across all studies, we measured pre-existing attitudes toward queer people and issues to assess their impact on researcher perceptions (see Supplemental Information for more information at https://osf.io/bdkre). Our results revealed that having more (vs. less) favorable pre-existing attitudes toward queer people were related to rating queer me-searchers as less biased and more informed. This is consistent with previous research (Altenmüller et al., 2021). However, the relationship between pre-existing attitudes and other outcome measures was not consistent across all studies, which may stem from the fact that we did not have sufficient power to test three-way interactions. Thus, while we recognize the importance of pre-existing attitudes, we believe that further—confirmatory—research is needed to clarify how these attitudes precisely relate to me-search stigma.

Our results pertaining to exploratory outcome measures suggest that me-search stigma is primarily linked to stereotypes about the researcher (e.g., personal benefit, appropriateness) rather than the perceptions of their work (quality of work and QRP ratings). Future work could explore these different stereotypes to determine whether they act as antecedents or outcomes of me-search stigma, using theory-driven predictions based on past theoretical (Jost & Van der Toorn, 2012) or empirical work (Thai et al., 2021). For instance, previous research has shown that perceptions of vested interest can undermine trust in science (Thai et al., 2021), suggesting that perceived personal benefit might serve as an antecedent to perceptions of bias. Conversely, perceptions of appropriateness could be a consequence of stigma, where the combined positive and negative stereotypes shape an overall assessment of the researcher’s “fit” for their work. Future studies could test these directional hypotheses with more robust experimental designs (Bullock & Green, 2021; Spencer et al., 2005).

We think it is important to emphasize the broader theoretical implications of this work. There is a growing body of research on public attitudes toward and trust in science (Većkalov et al., 2024, 2025) and scientists (Gligorić, Kleef, et al., 2024; Gligorić et al., 2025). However, this literature is usually silent on the role of scientists’ personal involvement in their research. Our findings, along with previous work on me-search (Thai et al., 2021) highlight the importance of examining the intersection of scientists’ identities and their work. With regards to advocacy, some emerging research on climate scientists suggests that they engage in more climate advocacy than researchers in other fields (Dablander, Sachisthal, Cologna, et al., 2024; Dablander, Sachisthal, & Haslbeck, 2024). As suggested by the results of Study 3, an advocacy-oriented approach is related to certain stereotypical perceptions of researchers (e.g., increased bias). Thus, the advocacy approach described as “trying to lead by example and address climate change beyond engaging in academic research” (Dablander, Sachisthal, & Haslbeck, 2024, p. 3) may unintentionally backfire and negatively influence attitudes toward climate science research. The solution is not to discourage advocacy but rather to further examine and understand how researchers and their work are perceived, taking into account the complex ways in which the two are intertwined.

### Practical Implications

Our findings, consistent with past empirical work (Altenmüller et al., 2021; Thai et al., 2021), support these anecdotal insights (Harrison & Michelson, 2022; Veldhuis, 2022), demonstrating me-search is stigmatized in the public’s eye. Some scholars have proposed that one way to mitigate the negative perception associated with me-search is to form mixed research teams (Altenmüller et al., 2021, p. 16). Yet, our findings show that allied researchers may also face a stigma for studying marginalized communities. Thus, the promise of mixed teams in reducing stigma around doing me-search remains an open empirical question.

Importantly, we believe that the responsibility for addressing this stigma lies more with institutions than with the individuals affected by it. For example, academic journals could begin by investigating if and how the review process differs for work by or focusing on marginalized communities compared to other research topics. Specifically, perception of bias might lead to harsher critique of methodologies or more frequent rejections based on *perceived* methodological flaws. By drawing on existing work, academic publishers and the broader academic community can develop strategies to combat the stigma associated with me-search and studies of marginalized communities, thereby fostering a more equitable research environment.

### Limitations and Future Directions

Our study has several limitations that warrant consideration, one being our subtle experimental manipulation of sexual orientation. We manipulated the sexual orientation of researchers by altering the names of the target researcher and their partners. We chose this approach because sexual orientation is not typically visible, making it challenging to manipulate through profile pictures (as in Thai et al., 2021). Moreover, explicitly stating the self-relevance of the research (as in Altenmüller et al., 2021) might inadvertently affect perceptions of the researcher’s motivation—which we aimed to manipulate independently (Study 3). Although we pretested these names for gender and other factors (see file “Pretesting Names”) and only included participants who correctly remembered the targets’ gender, more explicit manipulations may reduce the likelihood of participants misremembering this information, thus have a positive effect on observed effect sizes. We observed effects that varied from small to large. In contrast, past empirical work using more explicit measures observed descriptively larger effects on average on comparable outcomes (Devendorf et al., 2023; Thai et al., 2021), whereas work relying on a subtler vignette manipulation found effect sizes similar to ours (Altenmüller et al., 2021). Future research could thus employ more explicit manipulations, such as detailed information in a hypothetical application form (Moss-Racusin et al., 2012).

Our exploratory findings suggested that neither the researcher’s sexual orientation nor their research topic significantly impacted perceptions of the quality of their work or anticipated QRPs. This could suggest that me-search stigma primarily influences the evaluation of the researcher but not their work or how they do their work. Alternatively, it may reflect the limitations of our manipulation, which did not include sufficient information about the researcher’s work to allow participants to make informed judgments. Future studies could address this by presenting more detailed information, such as a research abstract, and manipulating relevant factors (Handley et al., 2015). This would offer a more rigorous test of whether me-search stigma primarily targets the researcher or extends to their work.

Additionally, our studies focused exclusively on how queer me-searchers are perceived relative to other queer and straight researchers. Similarly, previous research has either examined concealable stigmatized identities like sexual orientation (Altenmüller et al., 2021) or more visible ones such as like gender, race, or age (Handley et al., 2015; Thai et al., 2021). We recommend future research on me-search stigma considering both concealable and visible stigmatized identities in tandem. The concealability of marginalized group identity raises important questions about disclosure. For instance, a queer me-searcher may choose to reveal their identity (or not) during a conference presentation (Harrison & Michelson, 2022). Whether (the disclosure of) a concealable stigma affects the stereotypes associated with me-search stigma differently than visible sources of stigma is an open empirical question. The extensive body of work on concealable stigma and disclosure (Bry et al., 2017; Camacho et al., 2020; Chaudoir & Quinn, 2010) can be tremendously beneficial in drawing theory-driven predictions.

Finally, future work can examine the generalizability of our findings further by incorporating target researchers from universities outside of the United States or with non-English names (M. Harris et al., 2015) or by collecting data from more countries than just the United Kingdom (Farland-Smith, 2009)—as the perception of researchers may depend on a variety of culture-related factors (Gligorić, Clerc, et al., 2024). Additionally, even though the data quality on Prolific is usually high (Douglas et al., 2023), researchers could also consider collecting nationally representative data or try to utilize panel data to overcome the potential idiosyncratic effects that may result from collecting data from a self-selected subset of a population (Newman et al., 2021; Stritch et al., 2017).

## Conclusion

Despite facing stigmatization, researchers from marginalized groups are often driven to study and address the mistreatment of their communities. Our empirical findings reveal that me-searchers are stereotyped as more biased when researching bias against their ingroup, whether or not their work explicitly focuses on social justice implications. Crucially, this stigma also affects potential straight allies who are perceived as less informed when investigating anti-queer bias. This complex interplay of stereotypes illustrates how research with social justice implications can be stigmatizing for both marginalized researchers and their allies, underscoring the need for more inclusive and supportive research environments.

## Appendix A

These are the forum posts used across three studies. We present the parts that vary across conditions within brackets.

### A.1. Example Forum Post From Study 1

“Hi all! This is my first post. My name is [Jennifer/Daniel] Miller, I’m a second-year Psychology graduate student at the University of Delaware. I got my undergraduate degree from the University of Iowa in 2018 and then worked for a few years as full-time research assistant before I applied to graduate school. Now I am working in the LGBTQ+ Bias and Social Perception Laboratory here at University of Delaware under the direction of my advisor.

I am interested in studying the processes responsible for the mistreatment of LGBTQ+ individuals. More specifically, my research will explore how bias against LGBTQ+ individuals can undermine the ways in which LGBTQ+ individuals are perceived, evaluated, and treated in a variety of different contexts, ranging from the workplace to the home.

I just moved to Delaware for my studies with my partner [Noah/Sophia]. Thankfully, [he/she] was able to find a job here as well. At the moment, me and my partner [Noah/Sophia] are looking for an apartment/studio to rent. Please let me know if you know any good areas! Looking forward to talking more!”

### A.2. Example Forum Post From Study 2

“Hi all! This is my first post. My name is Daniel Miller, I’m a second-year Psychology graduate student at the University of Delaware. I got my undergraduate degree from the University of Iowa in 2018 and then worked for a few years as full-time research assistant before I applied to graduate school. Now I am working in the [LGBTQ+ Bias/Attentional Bias] and Social Perception Laboratory here at University of Delaware under the direction of my advisor.

I am interested in studying the processes responsible for [the mistreatment of LGBTQ+ individuals/how we perceive other people]. More specifically, my research will explore how [bias against LGBTQ+ individuals can undermine the ways in which LGBTQ+ individuals/visual and attentional biases can undermine the ways in which people] are perceived, evaluated, and treated in a variety of different contexts, ranging from the workplace to the home.

I just moved to Delaware for my studies with my partner [Noah/Sophia]. Thankfully, [he/she] was able to find a job here as well. At the moment, me and my partner [Noah/Sophia] are looking for an apartment/studio to rent. Please let me know if you know any good leads! Looking forward to talking more!”

### A.3. Example Forum Post From Study 3

“Hi all! This is my first post. My name is Daniel Miller, I’m a second-year Psychology graduate student at the University of Delaware. I got my undergraduate degree from the University of Iowa in 2018, and then worked for a few years as full-time research assistant before I applied to graduate school. Now I am working in the Social Bias and Social Perception Laboratory here at University of Delaware under the direction of my advisor.

I am interested in studying the processes responsible for the mistreatment of LGBTQ+ individuals. More specifically, my research will explore how bias against LGBTQ+ individuals can undermine the ways in which LGBTQ+ individuals are perceived, evaluated, and treated in a variety of different contexts, ranging from the workplace to the home. (*Neutrality condition*: [In doing this research, I am driven by the theoretical implications of my work with a perspective rooted in neutrality. I approach the results of my studies mostly as theoretical contributions that help us all understand the world around us better. To be good scientists, I believe that it’s crucial for researchers to remain impartial when it comes to their results.]; *Advocacy condition*: [In doing this research, I am driven by the social justice implications of my work with a perspective rooted in advocacy. I approach the results of my studies mostly as practical contributions that help us make the world around us a better place for all. To be good scientists, I believe that it’s crucial for researchers to use their work to help fight for positive social change.]).

I just moved to Delaware for my studies with my partner [Noah/Sophia]. Thankfully, [he/she] was able to find a job here as well. At the moment, me and my partner [Noah/Sophia] are looking for an apartment/studio to rent. Please let me know if you know any good leads! Looking forward to talking more!”

## Notes

1. We are cognizant that using “queer me-searcher” as a shorthand may be confusing as “me-searchers” study a variety of topics. One could also argue that an overwhelming majority of psychological research is me-search as it relies heavily on Eurocentric schools of thought and recruits White Western-European and North American samples (Ray, 2016). That said, in the current paper, we decided to use “queer me-searcher” for the sake of brevity as we focus on researchers studying anti-queer bias or attentional bias. Thus, in this context, there is only one possible “queer me-searcher.”

2. One might question excluding participants based on manipulation checks, but our items primarily checked whether participants correctly identified and remembered the manipulation texts. However, we still ran our confirmatory tests on the full pre-registered sample (*n* = 339), which yielded the same results in both significance and direction (see “Results full sample.pdf” at https://osf.io/s72yw).

3. Besides this sample, we also conducted the confirmatory tests with the sample without excluding participants based on manipulation checks (*N* = 380). The results remained the same both in terms of statistical significance and the direction of the observed effects. These results can be found in the supplementary materials (file titled “Results full sample” at https://osf.io/s72yw).

4. We pre-registered to collect data from 300 participants (for the power analysis see https://osf.io/8h47r/) and after doing so we had to exclude more than 100 participants who failed the manipulation checks. A big chunk of these participants was in the neutrality condition. Thus, we collected some additional data to meet our pre-registered sample size and oversampled the *neutral approach* condition to reach a relatively equal distribution across four conditions.

5. We have made an error here and deleted the third attention check while finalizing our survey. We checked if any participant failed both attention checks instead of 2 out of 3.
